# Antibacterial activities of titanium dioxide (TiO_2_) nanotube with planar titanium silver (TiAg) to prevent orthopedic implant infection

**DOI:** 10.1186/s13018-024-04596-0

**Published:** 2024-02-16

**Authors:** Hongli Zhang, Zhihui Jin

**Affiliations:** 1 Department of Surgery, Wuhan Fourth Hospital, Wuhan, 430030 Hubei Province China; 2 Department of Orthopaedics, Renmin Hospital of Wuhan University, Wuhan, 430060 Hubei Province China

**Keywords:** TiO_2_ nanotubes, Orthopedic implant infection, Orthopedic specialist QoC, Planar TiAg, Antibacterial properties

## Abstract

**Background:**

Orthopedic implant infection has become a common catastrophic complication after various orthopedic implants, which can lead to prolonged use of antibiotics and even surgical failure. The quality of care (QoC) of orthopedic implant infection is very important.

**Methods:**

Titanium dioxide (TiO_2_) nanotube array with planar TiAg was prepared, and their antibacterial rates were tested. 400 patients hospitalized in the Department of Orthopedics of Wuhan Fourth Hospital from May 2019 to May 2020 were selected as controls (before QoC evaluation system of orthopedics), and 400 patients hospitalized from June 2020 to June 2021 were selected as observation group (after QoC evaluation system of orthopedics).

**Results:**

Regardless of *Staphylococcus aureus* or *Escherichia coli*, the antibacterial rate of TiO_2_ nanotube array with planar TiAg was clearly higher than that of pure iron film on the 10th and 20th days (*P* < 0.05). The accuracy of hospitalization assessment, disease assessment, adverse event intervention, nursing record filing and nursing satisfaction in observation group were higher as against controls (*P* < 0.05).

**Conclusion:**

The TiO_2_ nanotube array with planar TiAg has good antibacterial property, which can effectively prevent orthopedic implant infection. The construction of QoC evaluation system for orthopedic specialists can effectively improve the QoC of orthopedic specialists.

## Introduction

With the development of medical technology, the research and application of orthopedic surgery have become more and more extensive, among which orthopedic implants occupy a very important position in clinical surgery. From limb fractures in trauma orthopedics and joint replacement in joint surgery, to osteotomy, orthopedic fusion, and fixation in spine surgery, and even fixation after resection of various bone tumors in the whole body, all of them are inseparable from internal fixation [1–3]. The main role of implants is to maintain the mechanical stability of bone after anatomical or functional reduction and to replace sick bone, to eliminate pain and provide good mechanical conditions for fracture healing and bone fusion. With the rapid development of orthopedics and its subspecialties, orthopedic implants are becoming more widely used [4]. In order to further improve the bone-promoting and anti-infection properties of implants, related research on implant coatings has become a hot topic. However, orthopedic implant infection has become a common catastrophic complication after various orthopedic implants, which can cause delayed bone healing or even nonunion, implant loosening, and further prolong of the use of antibiotics and even surgical failure [5]. Wildemann et al. [6] suggested that the introduction of implants alters the original tissue structure and physiological environment, leading to a weakened defense capacity of the local immune system against external microorganisms. The implant surface provides a foundation for bacterial adhesion, thereby increasing the risk of infection. Additionally, orthopedic surgeries themselves may cause tissue damage and trauma, providing pathways for bacterial invasion. Moreover, instruments and procedures during surgery may introduce exogenous bacteria, further raising the probability of infection. The implant surface is often a vulnerable area lacking antimicrobial barriers, and the smoothness and biocompatibility of biomaterials and metal implants allow bacteria to easily adhere and form biofilms, resisting attacks from the immune system. Consequently, research on orthopedic implant infections primarily focuses on developing antimicrobial surfaces and new anti-infection strategies to minimize the risk of infection.

As one of the three great inventions in the twenty-first century, nanomaterials have become the focus of attention in many fields, providing a lot of new development space for chemistry, materials, physics, and biology [7]. Compared with ordinary TiO_2_, nano-TiO_2_ has better chemical stability, thermal stability, dispersibility, non-migration, hydrophilicity, weather resistance, antibacterial self-cleaning, ultraviolet protection, and other characteristics, which can be applied in a wider range. It has a strong substitution for ordinary TiO_2_ in high-end application fields and special application fields [8–10]. Nano-TiO_2_ has excellent antibacterial self-cleaning function, strong antibacterial ability, broad antibacterial spectrum, long antibacterial effect, non-toxic and safe characteristics and can be used to manufacture antibacterial fibers, antibacterial antifouling coatings, antibacterial fluorescent lamps, and other products [11]. Nano-TiO_2_ has excellent UV protection performance, which can absorb, reflect, and scatter UV through visible light. It is a kind of physical shielding UV protective agent. Due to its natural whiteness, it can be adopted to produce sunscreen cosmetics and can also be used to produce photoaging-resistant coatings, plastics, inks, and other products. In addition, nano-TiO_2_ can also be adopted in environmental protection fields such as sewage treatment and gas purification, as well as self-cleaning glass production and solar cell manufacturing [12, 13].

Day care is a medical service model in which the whole process of inpatient diagnosis and treatment is completed within 24 h, which is a component of inpatient services. For example, day care ward, day treatment center, and other day care services, including day surgery and day chemotherapy, are implemented [14]. With the progress of medical technology and diagnosis and treatment concept, day care model represented by day surgery is developing rapidly around the world. Long-term practice has proved that it can effectively improve the efficiency of medical resources utilization. Nurse-Led Clinics (NLCs), as an advanced nursing practice model, is a nurse-led formal organized form of health care service delivery in the outpatient department. NLCs guide patients to master the self-care skills of specialized diseases and chronic diseases at home, and expand the continuous service from hospitalization to outpatient department and hospital to home, to meet the health service needs of patients and their families [15, 16]. At present, specialist nursing outpatient service is still in the preliminary exploration stage in China. How to improve the quality of specialist nursing and make it more scientific and efficient is a problem worth exploring.

In conclusion, as a common catastrophic complication after various orthopedic implants, it is of great significance to explore how to prevent and treat infection in orthopedic implants. Therefore, in order to solve the problem of anti-infection of orthopedic implants, TiO_2_ nanotube arrays with planar TiAg were prepared, and the antibacterial property of pure iron film deposited by pure iron was investigated as a control.

## Material and method

### Patients

400 patients hospitalized in the Department of Orthopedics of Wuhan Fourth Hospital from May 2019 to May 2020 were selected as controls (pre-QOC evaluation system of orthopedics department), and 400 patients hospitalized from June 2020 to June 2021 were selected as observation group (post-QOC evaluation system of orthopedics department) to evaluate the QoC of orthopedics specialty.

### Preparation of TiO_2_ nanotube arrays

The titanium plate for clinical use in orthopedics was cut into small pieces and polished repeatedly with gold photographic paper until the surface of the titanium plate was smooth and without scratch was observed under the scanning electron microscope. Then, the mixture of hydrofluoric acid and nitric acid with a ratio of 1:3 was passivated for 30 s, followed by ultrasonic cleaning with absolute ethanol, acetone, and double distilled water for 30 s, and then the mixture was removed and dried. The prepared electrolyte was put into a heat collector constant-temperature magnetic stirrer (Gongyi Yuhua Instrument Co., Ltd., China). The pretreated titanium plate as cathode in the pulsed DC magnetron sputtering ion plating equipment, the planar TiAg was used as anode in the silicon plate. Planar TiAg deposition with a diameter of 3 mm silver wire was adopted to obtain the TiAg composite film, a pure iron film of pure iron circular deposition as a control. The anode and cathode were immersed into the electrolyte, the distance between the electrodes was 2 cm, the temperature was set at 20 °C, the voltage was set at 40 V, and the speed was set at 120 times /min to obtain the TiO_2_ nanotube array with planar TiAg.

### Preparation of bacterial suspension

*Escherichia coli* and *Staphylococcus aureus* were inoculated in the prepared solid medium (agar 20 g, beef paste 5 g, peptone 10 g, sodium chloride 5 g) at 37 °C for one day with shaking, and transferred once a day to activate the strain. The activated fresh bacteria were scratched and added to the liquid medium with the inoculation ring (Guangzhou Bio-Mark Biotechnology Co., Ltd., China) at 37 °C for 20 h, and diluted according to the national standard GB 4789.2-2010 operation method.

### Plate counting method

The antimicrobial properties of TiO_2_ nanotube array with planar TiAg were determined by plate counting. 50µL bacterial suspension with a concentration of 1 × 10^5^ cfu/mL was aspirated on the surface of the titanium plate with a pipetting machine (Dragon Laboratory Instruments Limited, Beijing), and the titanium plate samples were cultured in a constant-temperature incubator (37 °C, 5% CO_2_) for 15 h. The bacterial solution on the titanium plate was then washed with 3 mL of phosphate buffer solution (PBS), and the adherent bacteria were separated using an ultrasonic oscillator (Granbo Technology Industrial Shenzhen Co., Ltd., China). The number of bacteria surviving on the surface of titanium plate samples was determined by coating dilution method, and the antibacterial rate of titanium plate samples was calculated.

1 $${\text{Antibacterial}} \;{\text{rate}} = \frac{M - N}{N} \times 100$$

*M* represents the number of bacteria present in the TiO_2_ nanotube array, and *N* represents the number of bacteria present in the TiO_2_ nanotube array in controls.

### Fluorescence staining method

50µL bacterial suspension at a concentration of 1 × 10^5^ cfu/mL was aspirated on the surface of the titanium plate by a pipette gun, and the titanium plate samples were cultured in a constant-temperature incubator (37 °C, 5% CO_2_) for 15 h. The specimens were washed with PBS to remove the non-adherent bacteria. During the bacterial live/dead staining experiment, the Live/Dead BacLight viability kit was employed, comprising two fluorescent dyes, namely SYTO9 and propidium iodide (PI). SYTO9 induced green fluorescence in viable bacteria, while PI caused dead bacteria to emit red fluorescence. The staining procedure was conducted at room temperature under dark conditions, with the staining solution thoroughly covering the adherent bacteria in each group. After 15 min, the specimens underwent three rinses with sterile PBS to ensure the removal of unbound staining solution. Subsequently, observations were made using laser scanning confocal microscopy (CLSM) to compare the survival status of surface bacteria in each group.

### Study subjects

A total of 400 patients were selected as the control group, with another 400 patients assigned to the observation group. In the control group, there were 223 males and 177 females, aged between 20 and 60 years, with an average age of 47.22 ± 4.55 years. The average hospitalization duration was 10.73 ± 3.05 days. The disease distribution included 128 cases of knee osteoarthritis, 104 cases of avascular necrosis of the femoral head, 72 cases of intertrochanteric femur fracture, and 61 cases of knee ligament injury. Additionally, there were 35 cases of knee meniscus injury. In the observation group, there were 201 cases, with 199 males and an age range of 22–61 years, and an average age of 45.63 ± 6.08 years. The average hospitalization duration was 11.54 ± 2.73 days. The disease distribution included 135 cases of knee osteoarthritis, 100 cases of avascular necrosis of the femoral head, 79 cases of intertrochanteric femur fracture, and 59 cases of knee ligament injury. There were also 27 cases of knee meniscus injury (Table 1). There were no statistically significant differences in age, gender, hospitalization duration, and disease types between the two groups (*P* > 0.05).

**Table 1:** Contrast of general data between two groups

Indicators	Observation group (*n* = 400)	Controls (*n* = 400)	*P*
Average age (years)	45.63 ± 6.08	47.22 ± 4.55	3.723
Gender (cases)			0.062
Male	201	223	
Female	199	177	
Length of stay (days)	11.54 ± 2.73	10.73 ± 3.05	0.087
Type of disease (cases)			0.059
Osteoarthritis of the knee	135	128	
Necrosis of the femoral head	100	104	
Fracture of the femoral trochanter	79	72	
Knee ligament injuries	59	61	
Meniscal injuries of the knee	27	35	

### Evaluation method

Controls used traditional quality evaluation criteria, including pass rates of ward management, nursing report writing, disinfection management, basic knowledge master, skills master. The inspection was generally carried out by the head nurse and the quality inspection committee.

The QoC evaluation system of orthopedic specialty was adopted in observation group. Firstly, an expert group composed of the director of nursing department, head nurse, orthopedic specialist nurse, and nursing team leader was established to comprehensively evaluate the scientific rationality of each index. The evaluation content was divided into factor quality (the number of beds managed by nursing staff, the ratio of beds to clinical nurses), link quality (the accuracy of hospitalization diagnosis, the accuracy of disease assessment, the accuracy of adverse event intervention, and the accuracy of nursing record), and final quality (the satisfaction of patients with nursing, the incidence of nursing defects). The number of beds (< = 8) and the ratio of beds to clinical nurses (> = 1:0.4) were evaluated by head nurses through on-site investigation and clinical evaluation every week. The final quality was evaluated by the director of nursing department through medical record review, questionnaire survey, department report, and patient complaint.

### Statistical method

SPSS19.0 statistical software was adopted for data analysis. Measurement data were expressed as mean ± standard deviation (‾x ± s), and count data were expressed as percentage (%). Repeated measurement analysis of variance was adopted for contrast between groups, and two-way analysis of variance for contrast within group. A two-sided test with *P* < *0.*05 was considered statistically meaningful.

## Results

### Bacterial live/dead staining results

**Fig. 1:**
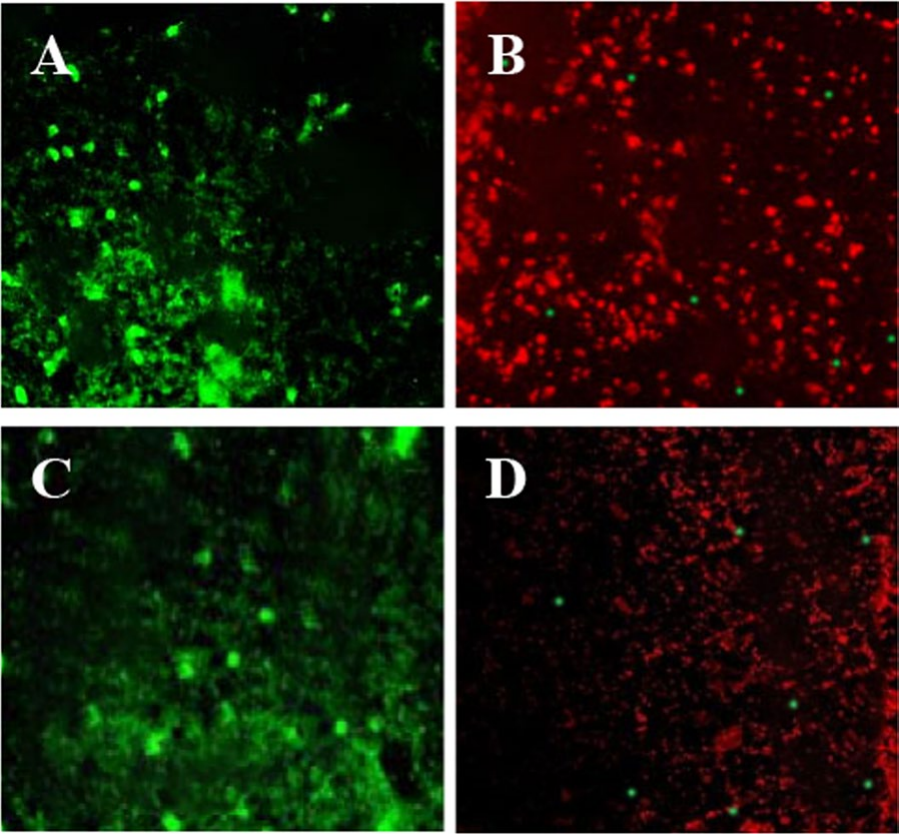
Bacterial live/dead staining results. *Note* **A** *Staphylococcus aureus* staining for the pure iron film group, **B** *Staphylococcus aureus* staining for the titanium dioxide nanotube array with a flat TiAg, **C** *Escherichia coli* staining for the pure iron film group, **D** *Escherichia coli* staining for the titanium dioxide nanotube array with a flat TiAg

After 48 h of constant-temperature static cultivation at 37 °C, staining observations were conducted on two groups: one with a flat TiAg surface of titanium dioxide nanotube array and the other with a pure iron film, using Staphylococcus aureus and Escherichia coli. As depicted in Fig. 1, the surface of the pure iron film group predominantly exhibited green fluorescence generated by viable bacteria, forming clustered aggregations and biofilm. In contrast, the surface of the titanium dioxide nanotube array with a flat TiAg mainly showed red fluorescence, with only isolated areas displaying green fluorescence. Furthermore, in this group, bacterial growth appeared more dispersed, lacking obvious clustering. This observation indicates a significant antibacterial effect of the titanium dioxide nanotube array with a flat TiAg against Staphylococcus aureus and Escherichia coli. It effectively inhibits their adhesion and proliferation on the coating surface while preventing the formation of biofilms.

### Antibacterial rate of TiO_2_ nanotube arrays

**Fig. 2:**
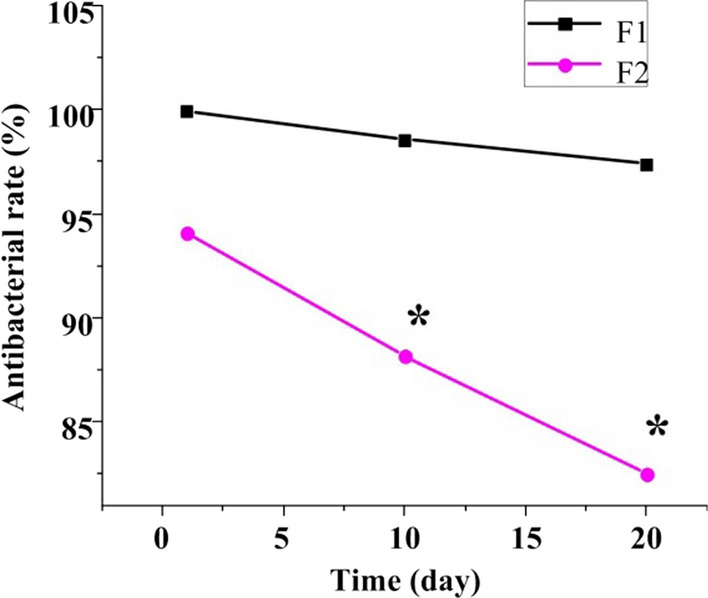
Contrast of antibacterial activity against Staphylococcus aureus. (F1: TiO_2_ nanotube array with planar TiAg; F2: pure iron film). *Note* * indicates that the difference is clear (*P* < 0.05)

Figure 2 shows the antibacterial rate of the TiO_2_ nanotube array with planar TiAg against *Staphylococcus aureus* was 99.91% on the first day, 98.55% on the 10th day, and 97.38% on the 20th day. The antibacterial rate of pure iron film on *Staphylococcus aureus* was 94.07%, 88.13%, and 82.51%, respectively. The antibacterial activity of the TiO_2_ nanotube array with planar TiAg against *Staphylococcus aureus* on the first day was not markedly different from that of the pure iron film (*P* > 0.05). The antibacterial activity of TiO_2_ nanotube array with planar TiAg against *Staphylococcus aureus* was higher as against pure iron film on days 10 and 20 (*P* < 0.05).

**Fig. 3:**
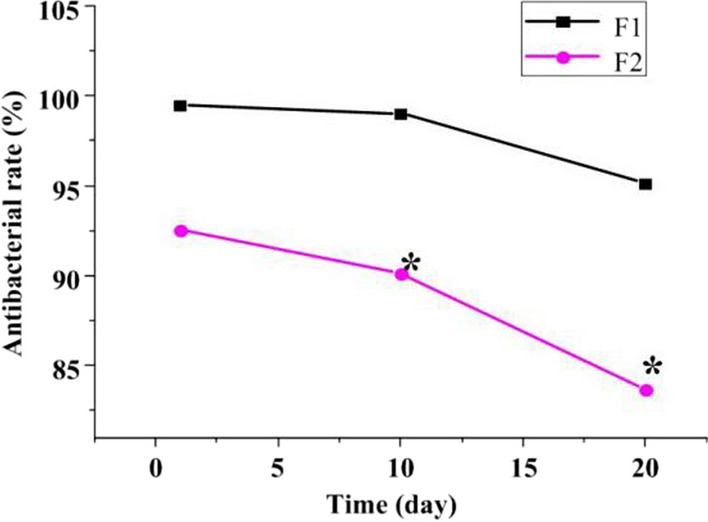
Contrast of the antibacterial activity against *E*. *coli*. (F1: TiO_2_ nanotube array with planar TiAg; F2: pure iron film)

Figure 3 reveals the antibacterial rate of the TiO_2_ nanotube array with planar TiAg against *E*. *coli* was 99.47% on the first day, 99.02% on the 10th day, and 95.13% on the 20th day. The antibacterial rate of pure iron film against *E*. *coli* was 92.54%, 90.11%, and 83.65%, respectively. The antibacterial activity against *E*. *coli* on the first day was similar (*P* > 0.05). The antibacterial activity of the TiO_2_ nanotube array with planar TiAg against *E*. *coli* was higher relative to pure iron film on days 10 and 20 (*P* < 0.05).

### Comparison of QoC indexes

**Fig. 4:**
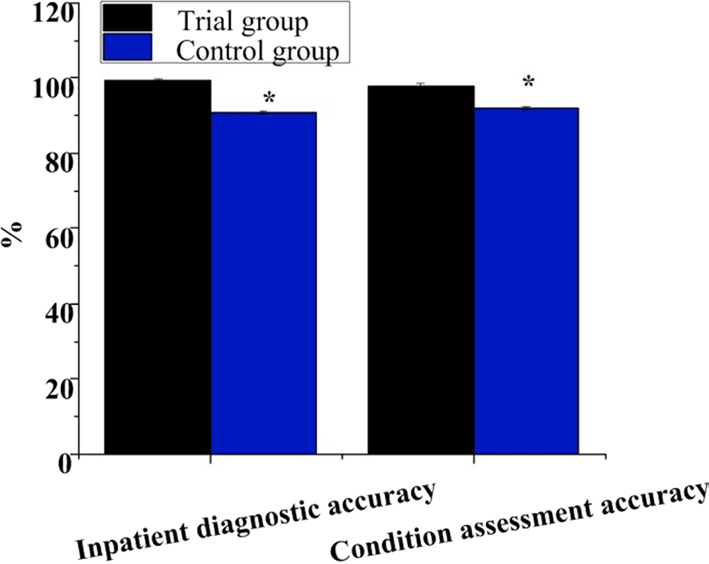
Contrast of the accuracy of hospitalization assessment and condition assessment. *Note* **A** is hospitalization assessment; **B** is condition assessment. * indicates *P* < 0.05 between observation group and controls

As illustrated in Fig. 4, the accuracy rate of hospitalization assessment in observation group was 99.35 ± 0.38%, and that of condition assessment was 98.04 ± 0.62%. They were 90.76 ± 0.45% and 92.16 ± 0.47% in controls. The accuracy of hospitalization assessment and disease assessment in observation group was superior as against controls (*P* < 0.05).

**Fig. 5:**
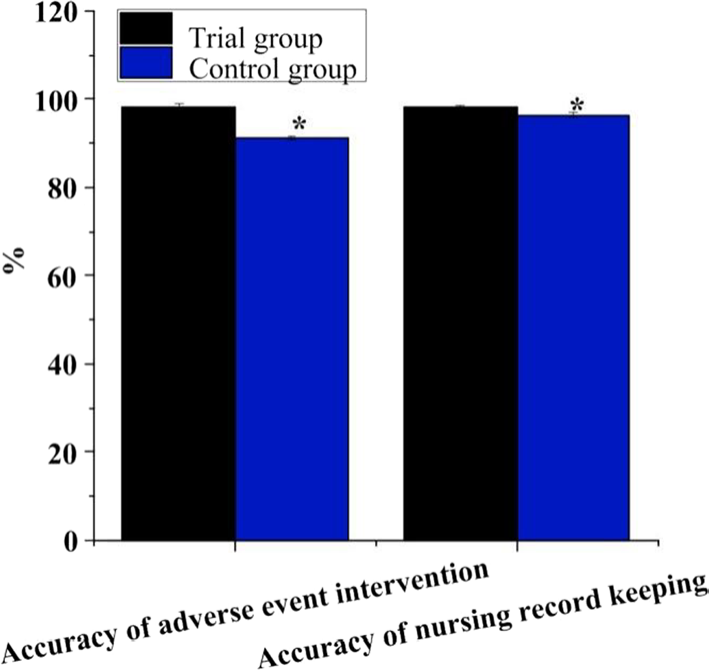
Contrast of the accuracy of adverse event intervention and nursing record filing. *Note* **A** is adverse event intervention; **B** is nursing record filing

Figure 5 illustrates the accuracy of adverse event intervention in observation group was 98.33 ± 0.71%, and that of nursing record filing was 98.06 ± 0.54%; they were 91.36 ± 0.38% and 86.43 ± 0.61% in controls. The accuracy of adverse events intervention and nursing record filing in observation group was superior in contrast to controls (*P* < 0.05).

**Fig. 6:**
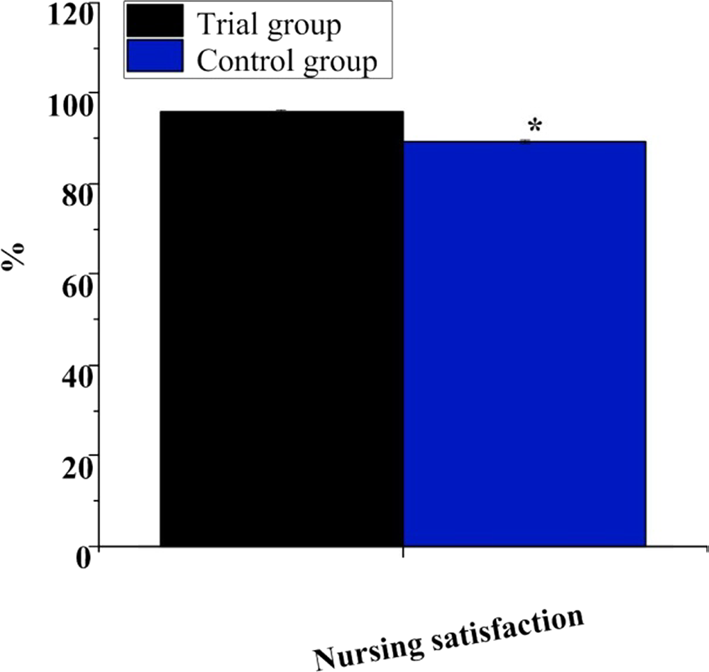
Contrast of the nursing satisfaction

Figure 6 indicates the nursing satisfaction of patients in observation group was 95.82 ± 0.56%, and that of controls was 89.36 ± 0.45%. The nursing satisfaction of observation group was higher as against controls (*P* < 0.05).

## Discussion

Orthopedic surgical infections have consistently posed a significant challenge in clinical treatment, drawing widespread attention due to their adverse effects on patient health. Surgical infections can lead to delayed healing, increased postoperative complications, and a decrease in the quality of life for patients. Confronted with this challenge, the search for effective anti-infection measures has become a crucial focus in orthopedic research [17, 18]. In this context, we aimed to address the issue of orthopedic surgical infections by selecting TiO_2_ nanotubes as the subject of our study. Introducing a titanium dioxide nanotube array with a flat TiAg surface, our goal was to explore a more efficient antimicrobial material. The primary objective of this study is to assess the antibacterial effectiveness of the titanium dioxide nanotube array with a flat TiAg surface based on TiO_2_ nanotubes in the treatment of orthopedic surgical infections. As a control, we used a pure iron film, intending to compare the antimicrobial properties of the two and validate the practical application potential of the novel nanotube array [19, 20].

The observational results indicate a significant antibacterial effect of the titanium dioxide nanotube array with a flat TiAg surface against Staphylococcus aureus and Escherichia coli. In contrast, the pure iron film group exhibited pronounced green fluorescence, suggesting the presence of viable bacteria and the formation of aggregated biofilm. This differential observation may be related to the physical and chemical properties of the material surface. The nanotube array surface predominantly showed red fluorescence, and bacteria appeared in a dispersed state, suggesting an antibacterial effect of TiAg. The antibacterial mechanism of TiAg may involve the disruption of microbial cell membranes, leading to cell death. This antibacterial effect could be a strong support for future treatments of orthopedic surgical infections. Additionally, the observed ability of the nanotube array to effectively inhibit biofilm formation is crucial in preventing the development of infections. Biofilms are complex structures formed by microorganisms on surfaces, not only making infections more challenging to treat but also potentially causing chronic infections. Therefore, the ability of the titanium dioxide nanotube array with a flat TiAg surface to inhibit biofilm formation is positively significant for improving the success rate of orthopedic surgeries and reducing the risk of infections. Li et al. [21] explored a new strategy for preventing and treating implant-related infections. They prepared clindamycin-loaded titanium and confirmed the slow release of clindamycin from the titanium surface through high-performance liquid chromatography. In vitro experiments involved co-culturing different titanium materials with Staphylococcus aureus, and the effects on bacterial parasitism and biofilm formation were determined using plate assays and scanning electron microscopy. In in vivo experiments, the antibacterial ability of clindamycin-loaded titanium and its impact on bone healing were evaluated using a rat osteomyelitis model. The results showed that clindamycin-loaded titanium exhibited antibacterial activity both in vitro and in vivo, reducing microbial burden and promoting bone healing. In comparison with this study, both research projects focus on surface modification of materials, achieving prevention and treatment of infections by introducing antibacterial substances. This study emphasizes the antibacterial effect and inhibition of biofilm formation of the titanium dioxide nanotube array with a flat TiAg surface, demonstrating promising applications. In future clinical applications, these two surface modification strategies may offer new perspectives and options for the prevention and treatment of implant-related infections.

The experimental results further indicated that the accuracy of inpatient assessment and condition assessment in the observation group is significantly higher, reaching 99.35% and 98.04%, respectively, compared to 90.76% and 92.16% in the control group. This suggests a notable improvement in the accuracy of inpatient assessment and condition assessment for patients following the implementation of the new observation group nursing intervention strategy. It reflects the effectiveness of specialized orthopedic nursing methods, contributing to a more accurate evaluation of patients’ health status and treatment needs. Moreover, specialized orthopedic nursing methods help address adverse events in a more timely and accurate manner, ensuring the completeness of nursing records, which is crucial for enhancing medical quality and patient safety. In terms of nursing satisfaction, specialized orthopedic nursing methods positively impact patient experience and satisfaction. Patients may be more satisfied with receiving more accurate and timely care and interventions, aiding in their better understanding and involvement in the treatment process, thereby improving the overall quality of medical services. A study conducted by Wang et al. aimed to explore the impact of high-quality care on orthopedic trauma [22]. Conclusions drawn from the experiments indicated that a high-quality orthopedic care model contributed to shortened recovery time, reduced pain intensity, and decreased analgesic time. Simultaneously, it facilitated patient recovery, reduced hospitalization duration, and improved quality of life and satisfaction. Both this study and Wang et al.’s research focus on the impact of nursing models on postoperative outcomes for orthopedic surgery patients, emphasizing the positive effects of new nursing models in reducing recovery time, alleviating pain, and lowering the incidence of complications. In summary, the results of both studies highlight the benefits of adopting new nursing models, particularly in orthopedic surgery patients. The introduction of biochemical indicators in Wang et al.’s study adds depth, while the commonality between the two articles lies in emphasizing the positive influence of high-quality care models on postoperative outcomes. The findings of this study suggest that specialized orthopedic nursing methods have a significantly positive impact on improving assessment accuracy, adverse event intervention effectiveness, accuracy of nursing record archiving, and patient satisfaction. This not only enhances the quality of medical services but also strengthens patient trust in the healthcare team. Such practical outcomes hold positive implications for improving the overall efficiency of medical institutions and the quality of patient care. Future research could provide more detailed descriptions of experimental details, especially standardizing experimental procedures and laboratory methods to ensure experiment reproducibility. Additionally, considerations for increasing the sample size and conducting multicenter studies could enhance the representativeness and generalizability of the research. Strengthening the review of relevant literature would offer a more comprehensive introduction to previous studies, providing better background and theoretical foundations for this research.

## Conclusion

In order to solve the problem of anti-infection in orthopedics, a TiO_2_ nanotube array with planar TiAg was prepared, and its antibacterial property was investigated. Moreover, the hospitalized patients in the Department of Orthopedics of Wuhan Fourth Hospital were enrolled to evaluate the QoC of orthopedics. The results reveal that the TiO_2_ nanotube array with planar TiAg has good antibacterial properties and can effectively prevent the problem of orthopedic implant infection. The construction of QoC evaluation system in orthopedics can make up for the lack of quality control of the traditional evaluation system and effectively improve the QoC of orthopedics. However, this paper has not carried out in vivo experiments for the TiO_2_ nanotube array with planar TiAg, which has not yet proved whether its antibacterial performance is as good in vivo. Therefore, TiO_2_ nanotube array will be applied to animal or clinical experiments in the following research. In conclusion, the results provide a reference for the prevention and treatment of implant infection and the evaluation of QoC in orthopedics.

## Data Availability

The original contributions presented in the study are included in the article.
