# Correction: Antibacterial activities of titanium dioxide (TiO_2_) nanotube with planar titanium silver (TiAg) to prevent orthopedic implant infection

**DOI:** 10.1186/s13018-025-05951-5

**Published:** 2025-06-17

**Authors:** Hongli Zhang, Zhihui Jin

**Affiliations:** 1 Department of Surgery, Wuhan Fourth Hospital, Wuhan, 430030 Hubei Province China; 2 Department of Orthopaedics, Renmin Hospital of Wuhan University, Wuhan, 430060 Hubei Province China

**Correction: Journal of Orthopaedic Surgery and Research (2024) 19:144**

10.1186/s13018-024-04596-0

In this article the author’s name Hongli Zhang was incorrectly written as Lihong Zhang.

The original article has been corrected.

